# Growth hormone excess drives liver aging via increased glycation stress

**DOI:** 10.18632/aging.206327

**Published:** 2025-10-03

**Authors:** Parminder Singh, Anil Gautam, Marissa N. Trujillo, Praveen Singh, Lizbeth Enriquez Najera, James J. Galligan, Lisa Hensley, Pankaj Kapahi, Andrzej Bartke

**Affiliations:** 1 Buck Institute for Research on Aging, CA 94945, USA; 2 Department of Internal Medicine, Southern Illinois University School of Medicine, Springfield, IL 62702, USA; 3 Department of Pharmacology and Toxicology, College of Pharmacy, University of Arizona, Tucson, AZ 85721, USA

**Keywords:** aging, growth hormone, glycation stress, Gly-Low

## Abstract

Growth hormone (GH) plays a crucial role in various physiological functions, with its secretion tightly regulated by complex endocrine mechanisms. Pathological conditions such as acromegaly or pituitary tumors result in elevated circulating GH levels, which have been implicated in a spectrum of metabolic disorders, potentially by regulating liver metabolism. In this study, we focused on the liver, a key organ in metabolic regulation and a primary target of GH, to investigate the impact of high circulating GH on liver metabolism. We used bovine GH overexpressing transgenic (bGH-Tg) mice to conduct a comprehensive transcriptomic analysis of hepatic tissues. The bGH-Tg mouse livers exhibit dysregulated fatty acid metabolism and heightened inflammatory responses. Notably, the transcriptomic profile of young bGH-Tg mouse livers resembled that of aged livers and displayed markers of increased cellular senescence. Furthermore, these mice exhibited a significant accumulation of advanced glycation end products (AGEs). Intervention with glycation-lowering compounds effectively reversed the insulin resistance and aberrant transcriptomic signatures in the liver that are associated with elevated GH levels. These findings underscore the potential therapeutic value of glycation-lowering agents in mitigating the deleterious effects of chronic GH overexpression.

## INTRODUCTION

Growth hormone (GH) is a pivotal endocrine regulator of growth and metabolism, orchestrating a range of physiological functions, including protein synthesis, lipolysis, and glucose homeostasis [1–3]. The secretion of GH is tightly controlled by a complex interplay of hormonal signals from the hypothalamus and pituitary gland, as well as feedback mechanisms involving insulin-like growth factor 1 (IGF-1) [4]. Dysregulation of GH secretion can lead to pathological conditions such as acromegaly and pituitary adenomas, characterized by excessively high circulating GH levels [5, 6]. These conditions have been linked to an array of metabolic disturbances, including insulin resistance, dyslipidemia, and increased cardiovascular risk.

GH, which profoundly influences the liver, is a central organ in metabolic homeostasis, directly modulating gluconeogenesis, fatty acid oxidation, and IGF1 production [1, 7]. However, the specific molecular mechanisms by which elevated GH levels impact liver metabolism and contribute to hepatic pathologies remain incompletely understood. Previous studies have suggested that chronic GH overexpression can induce hepatic steatosis and inflammation, yet the detailed transcriptomic changes have not been fully elucidated.

To address these gaps, we utilized bovine growth hormone transgenic (bGH-Tg) mice, a model for chronic GH overexpression. By performing comprehensive transcriptomic analyses of liver tissues from these mice, we aimed to elucidate the effects of high circulating GH levels on hepatic metabolism and inflammatory responses. Our findings indicate that bGH-Tg mice exhibit significant perturbations in fatty acid metabolism and activation of inflammatory pathways. Remarkably, the liver transcriptomic profile of young bGH-Tg mice mirrors that of the aged liver, with pronounced signs of cellular senescence and AGE accumulation. The accumulation of AGEs, known to be associated with various chronic diseases, suggests a potential therapeutic target. AGEs are formed through non-enzymatic glycation of proteins and lipids, leading to altered cellular function and signaling. Given the observed accumulation of AGEs in bGH-Tg mice, we investigated the therapeutic potential of glycation-lowering compounds. Our results demonstrate that treatment with these compounds can reverse the deleterious transcriptomic changes in the liver and mitigate multiple pathologies associated with elevated GH levels.

In this study, we provide a detailed analysis of the molecular changes induced by high circulating GH in the liver. We propose glycation-lowering interventions as a promising strategy to combat the associated metabolic disorders. These findings contribute to a deeper understanding of the hepatic effects of GH and highlight novel therapeutic avenues for conditions characterized by GH dysregulation.

## RESULTS

### Effect of GH overexpression on the liver transcriptome

**Figure 1:**
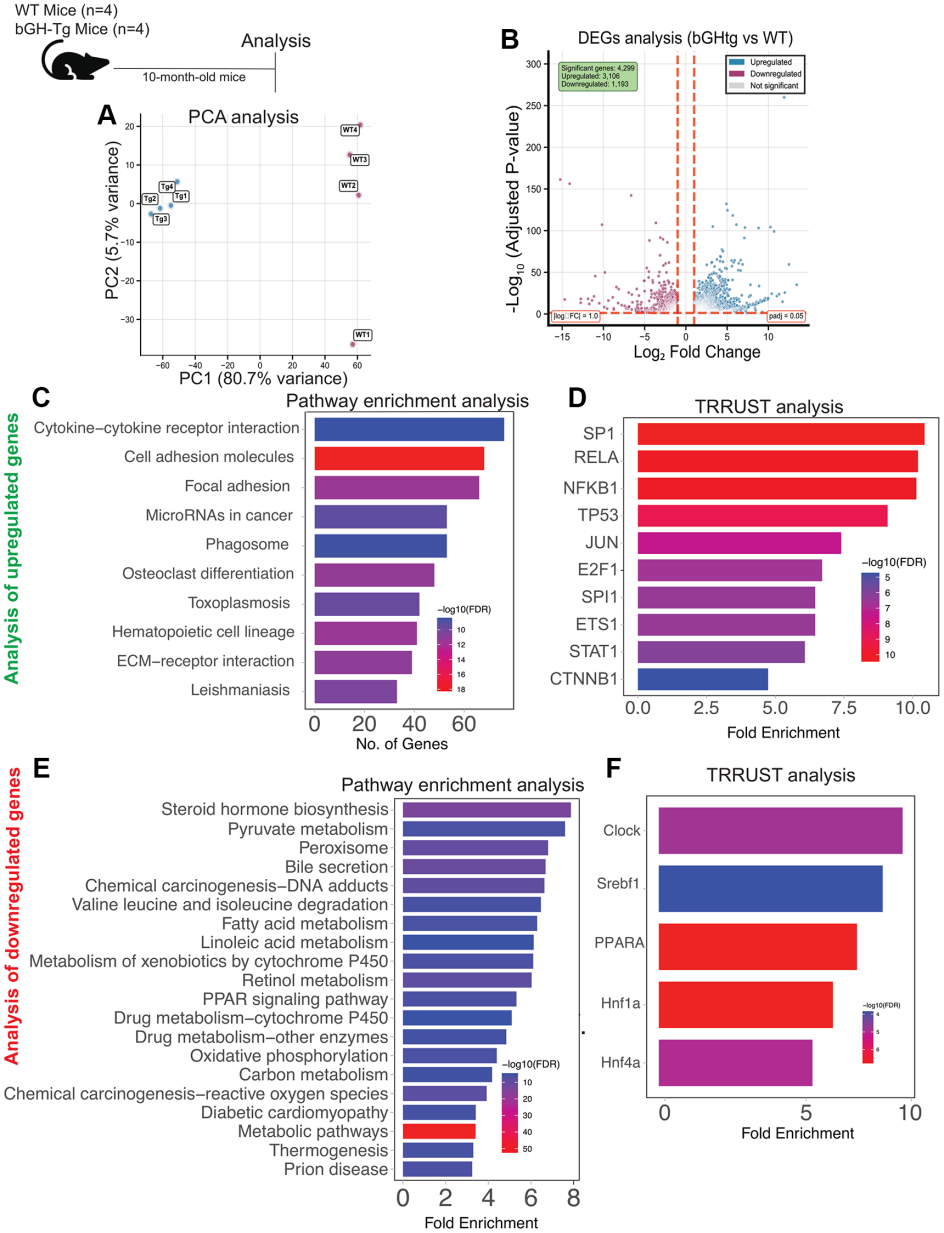
**Transcriptomic alterations in the liver of bGH-Tg mice compared to WT controls.** Three-month-old bGH-Tg and WT (C57BL6J) male mice were used for this study, and liver tissues were collected at 10 months of age for bulk RNA-seq analysis. Principal component analysis (**A**) shows clear segregation between WT and bGH-Tg liver transcriptomes. Differential expression analysis (**B**) identifies 4,209 upregulated and 1,193 downregulated genes in bGH-Tg mice compared to WT controls. Pathway enrichment analysis of upregulated genes (**C**) reveals enrichment of cytokine–cytokine receptor interaction, cell adhesion molecules, focal adhesion, microRNAs in cancer, hematopoietic cell lineage, and ECM–receptor interaction pathways, consistent with inflammatory and immune activation. TRRUST analysis (**D**) implicates transcription factors including SP1, RELA, NFKB1, TP53, JUN, and STAT1 as potential regulators of these pathways. Analysis of downregulated genes (**E**) highlights enrichment of metabolic and detoxification pathways, including steroid hormone biosynthesis, pyruvate metabolism, bile secretion, fatty acid metabolism, PPAR signaling, oxidative phosphorylation, and xenobiotic metabolism by cytochrome P450. TRRUST analysis (**F**) further identifies transcription factors such as CLOCK, Srebf1, PPARA, and Hnf family members as regulators of these metabolic changes, underscoring disrupted metabolic homeostasis in bGH-Tg livers.

Previous studies have established that chronic exposure to elevated growth hormone (GH) levels can precipitate hepatic and other pathology in bGH-Tg mice [8–10]. To elucidate the molecular underpinnings of GH-induced liver dysfunction, we conducted bulk RNA sequencing of liver tissues from bGH-Tg mice and age-matched wild-type (WT) controls. Our analysis identified significant segregation and dysregulation in gene expression, with 1193 genes downregulated and 3169 genes upregulated in bGH-Tg mice compared to WT controls (Figure 1A, 1B).

Pathway enrichment analysis of the upregulated genes revealed a pronounced involvement in immune response and inflammatory processes. Specifically, pathways related to positive regulation of cytokine production, leukocyte activation, inflammatory response, platelet activation, endocytosis, extracellular matrix organization, and neutrophil degranulation were prominently affected (Figure 1C). These findings align with previous studies indicating that GH overexpression can lead to chronic low-grade inflammation in various tissues, including the liver [11–13]. TRRUST analysis implicated several transcription factors (TFs) as potential regulators of these pathways, including TRP53, NF-kB1 (NF-κB), IRF4, STAT1, and RFXAB, (Figure 1D) highlighting their role in orchestrating the inflammatory response in GH-overexpressing conditions [14–16].

Furthermore, we observed an upregulation of the p21-derived senescence signature in the liver of bGH-Tg mice compared to WT controls (Figure 1B and Supplementary Figure 1A, 1B). Cellular senescence, characterized by irreversible growth arrest and senescence-associated secretory phenotype (SASP), has been implicated in liver aging and dysfunction [17, 18]. The observed transcriptomic signature underscores the accelerated senescence and aging phenotype associated with chronic GH exposure in the liver.

In contrast, pathway enrichment analysis of the downregulated genes highlighted perturbations in metabolic pathways critical for liver function. These included mono-carboxylic acid metabolic processes, bile secretion, fatty acid metabolism, lipid homeostasis, and PPAR signaling pathways (Figure 1E). Disruption of these pathways is consistent with previous reports linking GH excess to impaired lipid metabolism and hepato-steatosis [19]. TRRUST analysis identified key transcription factors such as SREBF1, CLOCK, and PPARA (PPAR-α) (Figure 1F) as potential mediators of these metabolic changes, suggesting their role in regulating lipid homeostasis under conditions of GH overexpression [20–24].

### Comparative transcriptional analysis of GH overexpression and aging effects on liver transcriptome

**Figure 2:**
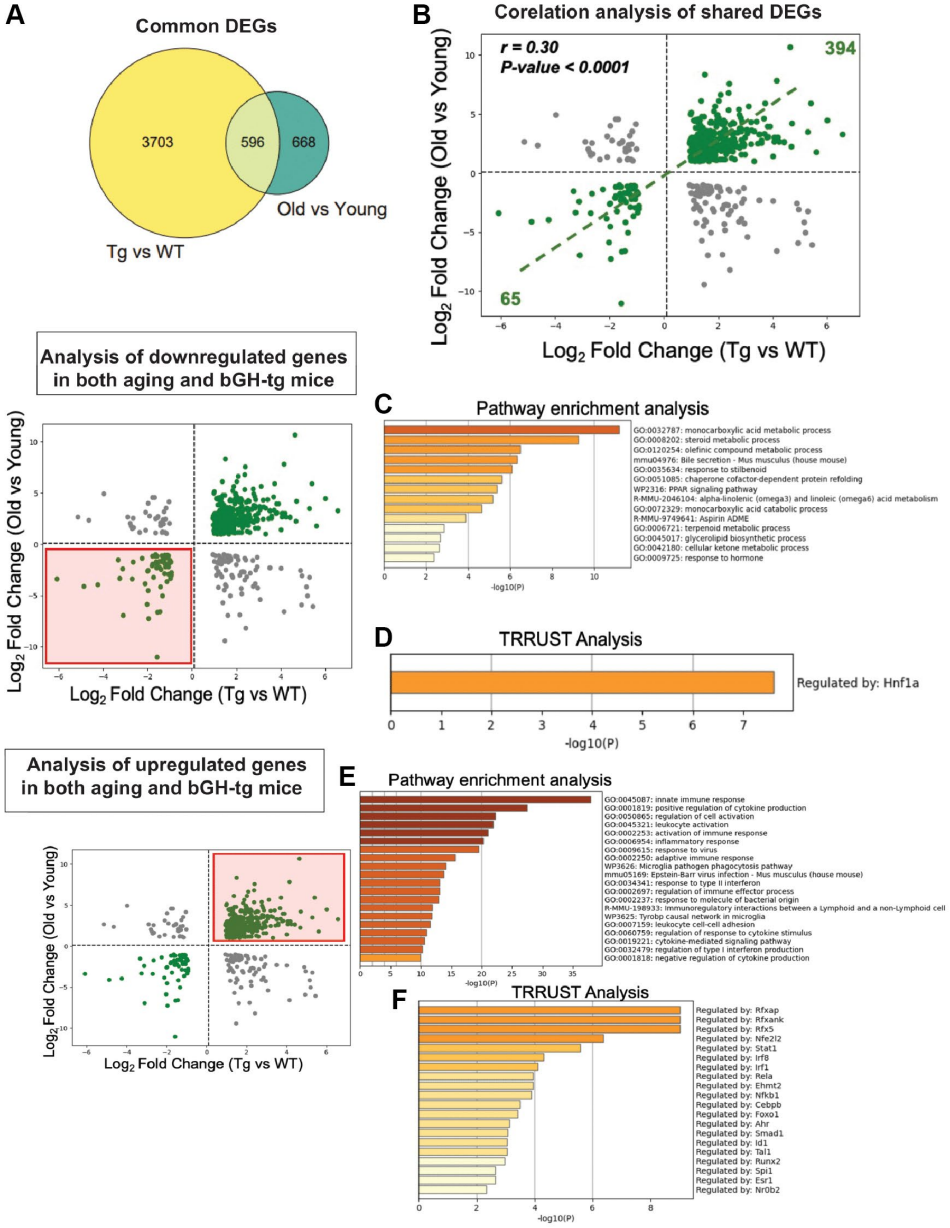
**Comparative transcriptomic analysis of GH overexpression and liver aging.** Differential gene expression profiles from bGH-Tg versus WT mice were compared with those from aged (24-month-old) versus young (10-month-old) WT mice to identify overlapping molecular signatures of GH-induced and age-associated changes. (**A**) Venn diagram showing 596 common differentially expressed genes (DEGs) shared between GH overexpression (Tg vs WT) and aging (Old vs Young). (**B**) Correlation analysis of shared DEGs reveals a significant positive correlation (r = 0.30, *p* < 0.0001), with 394 genes upregulated and 65 genes downregulated in both conditions. (**C**) Pathway enrichment analysis of shared downregulated genes demonstrates enrichment of key metabolic pathways. (**D**) TRRUST analysis of shared downregulated genes identifies Hnf1a as a major regulatory transcription factor. (**E**) Pathway enrichment analysis of shared upregulated genes highlights enrichment of key gene ontology pathways. (**F**) TRRUST analysis of upregulated shared genes implicates transcription factors including Rfxap, Stat1, Irf1, Nfkb1, Rel, and other immune regulators as key drivers of the inflammatory transcriptional program common to both GH overexpression and aging.

Given the striking similarities in pathologies observed between bGH-Tg mice and naturally aged animals, we next sought to directly compare the liver transcriptomic profiles of these two conditions. To this end, we analyzed differential gene expression profiles (DEGs) from young (10-month-old) bGH-Tg mice relative to age-matched WT controls, alongside DEGs derived from old (24-month-old) versus young (10-month-old) WT mice. This comparative approach revealed 596 genes that were commonly dysregulated in both datasets, representing a substantial overlap between GH-induced and age-associated transcriptional changes (Figure 2A). Importantly, correlation analysis of these shared DEGs demonstrated a significant positive correlation (r = 0.30, *p* < 0.0001), indicating that chronic GH excess drives transcriptional reprogramming that mirrors, at least in part, the molecular signature of liver aging (Figure 2B).

To further dissect the biological significance of these shared alterations, we separated the common DEGs into downregulated and upregulated subsets and performed functional enrichment analyses. The downregulated genes were strongly enriched for metabolic pathways central to hepatic function, including monocarboxylic acid metabolism, bile secretion, PPAR signaling, and ketone body metabolism (Figure 2C). These pathways play critical roles in energy homeostasis, lipid utilization, and detoxification, and their coordinated repression suggests that both GH overexpression and aging compromise fundamental aspects of hepatic metabolic capacity. Consistent with this, TRRUST analysis identified Hnf1a, a key hepatic transcription factor involved in metabolic regulation, as a putative upstream driver of these downregulated signatures (Figure 2D) [25, 26].

In contrast, the upregulated subset of shared DEGs revealed robust enrichment of immune- and inflammation-related pathways, including innate immune response, cytokine production, leukocyte activation, and antigen presentation (Figure 2E). This pattern indicates that both GH overexpression and aging promote a pro-inflammatory transcriptional landscape within the liver. TRRUST analysis highlighted several immune-associated transcription factors, including Nfe2l2, NF-κB1, RFXAP, and STAT1, as candidate regulators of these inflammatory networks (Figure 2F). Together, these results align with the concept of inflammaging, whereby persistent low-grade inflammation is a hallmark of aging tissues and is similarly exacerbated under conditions of GH excess [27].

Collectively, this dual signature—suppression of core metabolic pathways and activation of immune-inflammatory programs—underscores the complex interplay between GH signaling and liver aging. These findings suggest that chronic GH overexposure accelerates molecular features of hepatic aging through both metabolic decline and heightened inflammation, providing mechanistic insight into the reduced lifespan and age-associated pathologies observed in GH transgenic mice. Importantly, this overlap also identifies potential therapeutic targets, particularly at the interface of metabolism and immunity, for mitigating liver dysfunction in both aging and GH-driven disease states.

### Advanced glycation end products (AGEs) as downstream mediators of pathologies associated with GH overexpression

**Figure 3:**
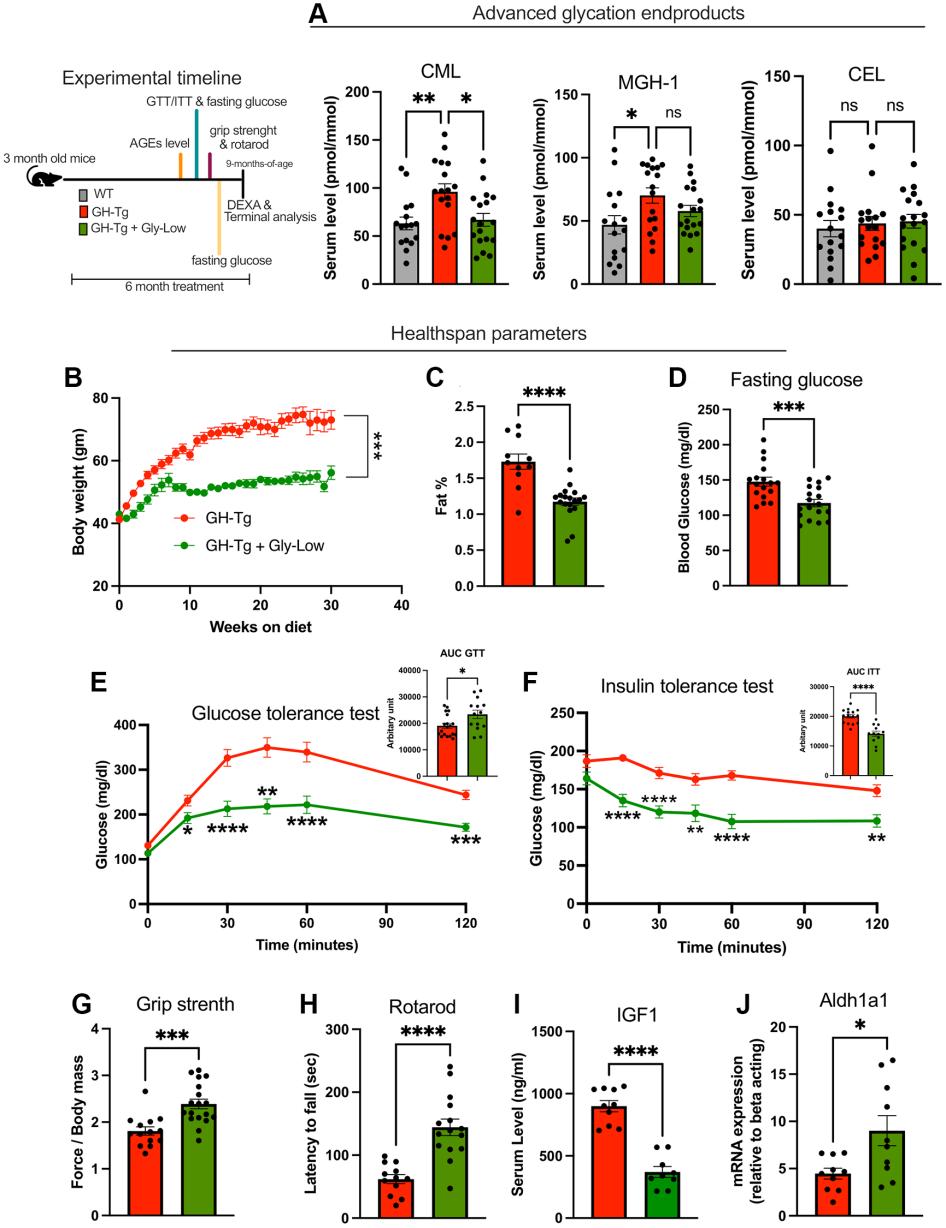
**Accumulation of advanced glycation end products (AGEs) and beneficial effects of Gly-Low treatment in bGH-Tg mice.** As shown in the experimental regime, 3-month-old WT, bGH-Tg, and bGH-Tg mice treated with Gly-Low were maintained on their respective diets for 6 months, after which animals underwent metabolic and functional assessments followed by terminal tissue collection. (**A**) Mass spectrometry–based quantification of serum AGEs revealed significantly elevated CML and MGH-1 in bGH-Tg mice compared to WT, with both reduced by Gly-Low treatment, while CEL levels remained unchanged. (**B**–**F**) Healthspan and metabolic parameters were assessed: Gly-Low–treated bGH-Tg mice exhibited lower body weight gain (**B**), reduced fat mass percentage (**C**), improved fasting glucose levels (**D**), enhanced glucose tolerance (**E**), and improved insulin sensitivity (**F**). (**G**, **H**) Functional testing demonstrated that Gly-Low improved grip strength and rotarod performance relative to untreated bGH-Tg mice. (**I**, **J**) Molecular analysis showed reduced circulating IGF1 and increased hepatic expression of Aldh1a1, a detoxification enzyme, in Gly-Low–treated bGH-Tg mice. Data are presented as mean ± SEM, and statistical significance is indicated by ^*^p ≤ 0.05, ^**^p ≤ 0.01, ^***^p ≤ 0.001, and ^****^p ≤ 0.0001.

Given the observed disruption in detoxification pathways in bGH-Tg mice, we hypothesized that the pathologies associated with GH overexpression may be partially mediated by the accumulation of molecular species that induce damage over time. One such molecular insult is the accumulation of advanced glycation end products (AGEs) in various tissues [28–30]. To assess AGE accumulation, we employed mass spectrometry-based analysis of liver and serum samples from bGH-Tg mice. Our analysis revealed a significant accumulation of various types of AGEs in transgenic mice, indicating heightened glycation-related stress in these tissues (Figure 3A–3C).

Given the pervasive role of AGEs in multiple age-related pathologies [28, 31–33], we hypothesized that the accumulation of AGEs contributes to the pathologies observed in bGH-Tg mice. To investigate this hypothesis, we treated 3-month-old bGH-Tg mice with our glycation-lowering cocktail (Gly-Low), previously demonstrated to selectively target glycation-induced stress in the body [34]. As expected, 5–6-month long treatment with Gly-Low led to rescue of circulating AGEs levels in the bGH-Tg mice (Figure 3A). Remarkably, we observed improvement of multiple health parameters in the Gly-low treated mice compared to the control treatment, specifically, Gly-Low-treated mice exhibited rescue in body weight gain characterized by reduced fat mass percentage (Figure 3B, 3C) while maintaining lean mass (data not shown). Additionally, these mice displayed lower fasting glucose levels compared to control mice after five months of treatments (Figure 3D), along with enhanced glucose tolerance (Figure 3E) and improved insulin sensitivity (Figure 3F). Moreover, functional assessments revealed that Gly-Low-treated mice exhibited improved motor coordination and muscle strength, as demonstrated by better performance in grip strength and Rota-rod assays compared to control mice (Figure 3G, 3H). Interestingly, molecular analyses of liver tissues from Gly-Low-treated mice showed decreased expression levels of IGF1, a marker associated with GH overexpression-induced pathologies (Figure 3I). Conversely, there was enhanced expression of aldh1a1 (Figure 3J), an enzyme involved in detoxification processes, suggesting a potential mechanism through which Gly-Low treatment mitigates the adverse effects of GH overexpression.

### Supplementation with Gly-Low partly reverses the transcriptomic signature in the liver induced by GH overexpression

**Figure 4:**
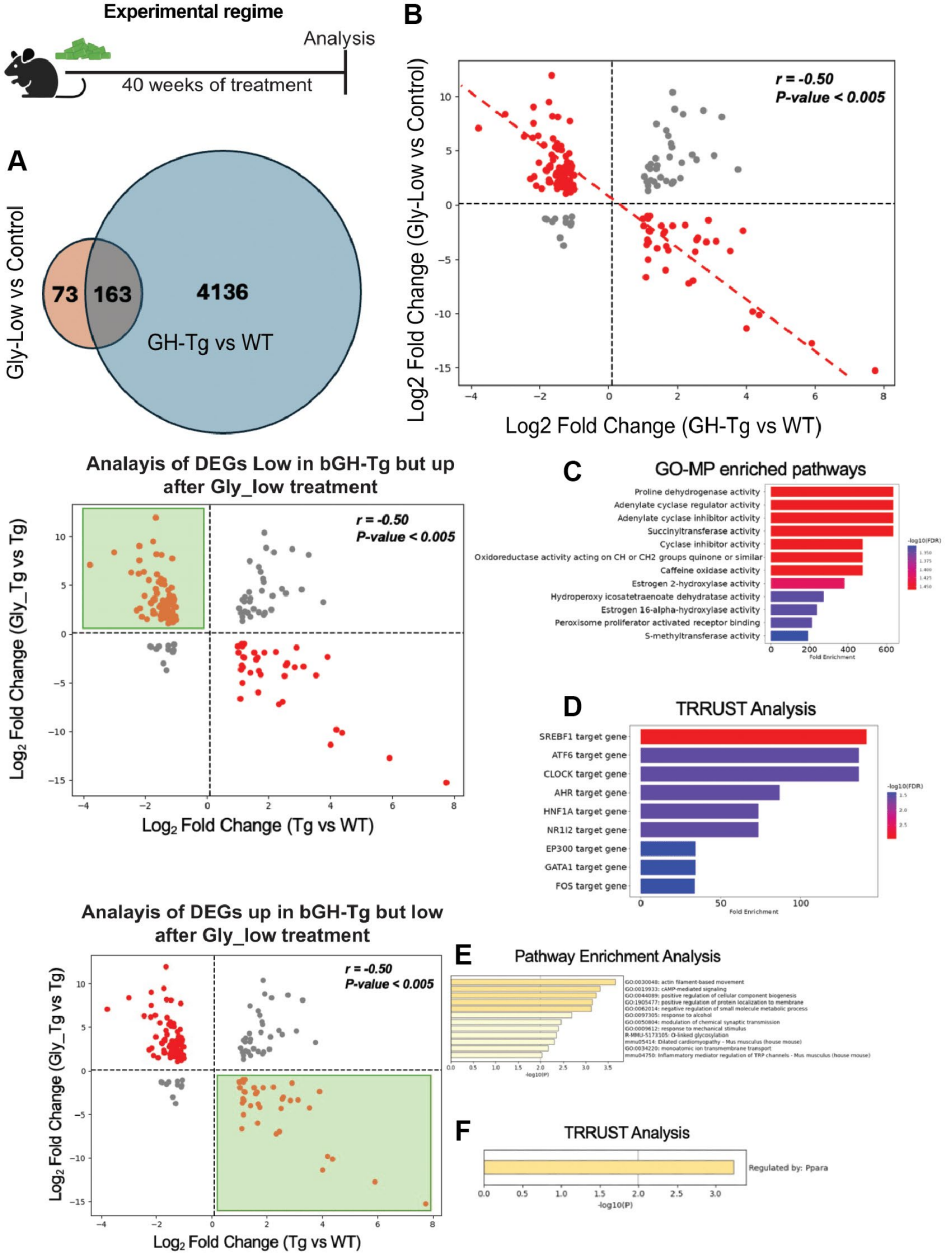
**Gly-Low treatment partially reverses transcriptomic alterations induced by GH overexpression.** As shown in the experimental regime, bGH-Tg mice were treated with Gly-Low or control diet for 40 weeks, after which liver transcriptomes were analyzed. (**A**) Venn diagram shows 163 differentially expressed genes (DEGs) overlapping between Gly-Low–treated versus control bGH-Tg mice and bGH-Tg versus WT comparisons. (**B**) Correlation analysis of shared DEGs demonstrates a strong negative correlation (r = –0.50, *p* < 0.005), indicating that Gly-Low treatment counteracts transcriptional changes driven by GH excess. (**C**, **D**) Analysis of genes downregulated in bGH-Tg mice but restored by Gly-Low revealed enrichment of pathways associated with oxidoreductase activity, adenylate cyclase regulation, estrogen metabolism, and PPAR signaling, with TRRUST implicating transcription factors including SREBF1, ATF6, CLOCK, HNF1A, and NR1I2 as regulators. (**E**, **F**) Conversely, analysis of genes upregulated in bGH-Tg mice but suppressed by Gly-Low treatment identified enrichment of pathways related to actin filament–based movement, cAMP-mediated signaling, and gluconeogenesis, with TRRUST highlighting PPARA as a key transcriptional regulator.

To further elucidate the molecular effects of Gly-Low treatment on liver transcriptome in bGH-Tg mice, we hypothesized that Gly-Low would mitigate AGE-induced glycation stress and consequently reverse some of the transcriptomic alterations induced by GH overexpression. To test this hypothesis, we performed the bulk RNAseq analysis of the liver from bGH-Tg mice treated with control and Gly-Low diet. Transcriptomic analysis revealed significant segregation of different groups with 235 differential expressed genes (Supplementary Figure 2A, 2B). Pathway enrichment and TRRUST analysis revealed Gly-Low may regulate key liver pathways such as gluconeogenesis and cAMP-mediated signaling potentially by modulating PPARA signaling (Supplementary Figure 2C, 2D). Next, we compared the differentially expressed genes (DEGs) from Gly-Low-treated transgenic mice (transgenic mice liver treated with Gly-Low vs. transgenic mice liver treated with a control diet) with DEGs identified in transgenic-induced changes (transgenic liver vs. WT liver). Our analysis revealed 163 shared DEGs between these datasets (Figure 4A). Notably, these shared genes exhibited a significant negative correlation (r = −0.50), suggesting that Gly-Low treatment partially reverses the transcriptomic changes induced by GH overexpression (Figure 4B).

Further analysis of genes that were downregulated in bGH-Tg mice and upregulated after Gly-Low treatment were associated with pathways involving proline dehydrogenase activity, adenylate cyclase activity, and estrogen 2-hydroxylase activity (Figure 4C). TRRUST analysis identified transcription factors such as SREBF1, ATF6 and CLOCK as key regulators of these processes (Figure 4D). SREBF1 and ATF6 are critical for lipid homeostasis and endoplasmic reticulum stress response, respectively [35, 36]. CLOCK, a core circadian regulator, has been shown to influence detoxification pathways and metabolic processes, indicating that modulation of circadian genes might be a viable strategy to combat glycation stress [37–39]. Conversely, pathway enrichment analysis of the genes upregulated in bGH-Tg liver, but downregulated following Gly-Low treatment, identified key pathways such as actin filament-based movement, cAMP-mediated signaling, response to alcohol, O-linked glycosylation, and inflammatory mediator regulation (Figure 4E). TRRUST analysis implicated PPARA as a potential regulator of these pathways (Figure 4F). The role of PPARA in lipid metabolism and inflammation is well-documented, suggesting that Gly-Low’s beneficial effects may be mediated through modulation of PPARA activity [23, 40].

To further validate these transcriptomic signatures, we complemented our systems-level analyses with targeted assays. Specifically, we investigated whether GH excess induces oxidative stress in the liver. Metabolomic profiling revealed that bGH-Tg mice exhibited a significantly reduced GSH/GSSG ratio and lower NADPH levels (Supplementary Figure 3A, 3B), consistent with increased oxidative stress and diminished antioxidant regeneration. Importantly, treatment with Gly-Low restored both redox parameters, supporting the conclusion that GH overexpression promotes oxidative stress, which can be alleviated by glycation-lowering interventions. At the protein level, we validated the expression of transcriptional regulators highlighted in our RNA-seq and TRRUST analysis. Western blotting demonstrated reduced protein levels of SREBF1, CLOCK, and PPAR-α in bGH-Tg livers, with PPAR-α expression rescued by Gly-Low treatment (Supplementary Figure 3C, 3D). These factors were selected for validation because they represent key nodes in lipid metabolism and circadian regulation identified by our transcriptomic data. Their altered expression provides direct protein-level support for our systems-level findings.

In summary, these findings indicate that Gly-Low partly counteracts GH-induced transcriptomic alterations, predominantly by reversing changes in key metabolic and inflammatory pathways. This underscores the therapeutic potential of targeting AGE-induced glycation stress and circadian gene modulation to mitigate pathologies associated with chronic GH overexpression.

## DISCUSSION

This study provides significant insights into the impact of chronic growth hormone (GH) overexpression on liver metabolism, highlighting key changes that may drive GH-induced metabolic disorders. Using a bovine GH-overexpressing transgenic (bGH-Tg) mouse model, we observed marked transcriptomic changes, highlighting dysregulation in fatty acid metabolism, heightened inflammatory responses, and evidence of cellular senescence within the liver, resembling aging profiles. These findings support the hypothesis that GH excess not only affects liver metabolism directly, but also accelerates hepatic aging processes, contributing to systemic metabolic dysregulation. The suggested acceleration of hepatic aging is consistent with the evidence that GH transgenic mice have markedly reduced longevity along with phenotypic traits that have been interpreted as symptoms of early aging [41, 42]. Interestingly, mice, in which GH signaling is suppressed rather than enhanced, are characterized by extended longevity and slower and/or delayed aging [43–46]. Our transcriptomic analysis revealed that GH overexpression perturbs immune and inflammatory pathways, corroborating previous findings linking GH excess to chronic low-grade inflammation. The enriched inflammatory pathways, including leukocyte activation and cytokine production, suggest that GH-driven inflammaging may be mediated by key transcription factors such as NF-kB and STAT1. This pattern of inflammatory upregulation parallels liver aging, suggesting that GH overexpression may act as an aging accelerant through sustained inflammation. Such a mechanism aligns with the concept of inflammaging, where chronic, low-grade inflammation contributes to metabolic and functional decline in aging tissues.

In parallel, GH overexpression disrupts metabolic pathways central to liver function, including fatty acid oxidation, bile secretion, and lipid homeostasis. These disruptions mirror changes observed in aged livers and underscore the liver’s sensitivity to GH dysregulation. Transcription factors such as PPAR-α, SREBF1, and CLOCK were implicated as central regulators of these pathways, highlighting their role in maintaining metabolic stability under normal conditions and their potential dysregulation in GH-induced hepatic stress. The overlap in transcriptional changes between bGH-Tg mice and aged livers further reinforces the idea that GH overexpression accelerates hepatic aging, likely through combined inflammatory and metabolic stress.

To further validate our transcriptomic findings, we performed targeted assays. Metabolomic profiling showed that bGH-Tg livers had a reduced GSH/GSSG ratio and lower NADPH levels, consistent with elevated oxidative stress, both of which were restored by Gly-Low treatment. At the protein level, Western blotting revealed reduced expression of SREBF1, CLOCK, and PPAR-α in bGH-Tg livers, with PPAR-α expression rescued by Gly-Low. These results provide direct functional support for our systems-level findings.

Another novel aspect of this study is the identification of advanced glycation end products (AGEs) as a prominent feature in the livers of bGH-Tg mice. The accumulation of AGEs, compounds linked to aging and metabolic diseases, suggests that GH-induced stress may overwhelm hepatic detoxification pathways, promoting glycation stress. AGEs are known to contribute to cellular dysfunction by altering protein structure and triggering inflammatory signaling, potentially exacerbating GH-driven liver pathologies. These findings position AGEs as not only a marker of GH-induced metabolic stress but also a possible mediator of GH-related liver dysfunction.

Our findings indicate that glycation-lowering compounds (Gly-Low) can mitigate many of the deleterious effects associated with GH overexpression. Gly-Low treatment reversed several GH-induced transcriptomic changes, particularly in pathways related to inflammation and metabolic homeostasis. Notably, Gly-Low downregulated inflammatory pathways and upregulated detoxification and lipid metabolic pathways, suggesting that glycation reduction can restore, at least partially, liver-mediated functions and glucose homeostasis disrupted by GH excess. This therapeutic approach has potential implications for managing conditions associated with chronic GH dysregulation, such as acromegaly, and may offer broader benefits for liver pathologies where glycation stress is prevalent.

While our systems-level approach provides a comprehensive framework, we recognize that additional mechanistic interrogation — particularly of NF-κB signaling and mitochondrial dysfunction — will be an important future direction.

In conclusion, this study underscores the profound impact of GH overexpression on liver aging and metabolic disorders, with AGEs identified as potential targets for therapeutic intervention. Glycation-lowering strategies may serve as effective treatments for alleviating GH-induced metabolic and inflammatory disruptions in the liver, offering a promising avenue for addressing age-related metabolic diseases associated with GH dysregulation.

## METHODS

### Animals

For this research, bovine GH transgenic mice were created in our colony from animals kindly donated by Dr. T. Wagner and J. S. Yun of Ohio University in Athens, Ohio [47]. Male transgenic mice were mated with normal (C57BL6/J × C3H/J F1) hybrid females to produce the mice. For this experiment, normal siblings of bGH Tg mice served as controls. Animals were kept in climate- and light-controlled environments (21–23°C with a 12-hour cycle of light and darkness). The Institutional Animal Care and Use Committee at Southern Illinois University approved all animal methods used in this investigation. The total number of mice used in this study was around 150, i.e., 15–20 mice per group.

### Weaning and genotype confirmations

All animals were weaned 21–22 days after birth and housed in a cage of 4–5 animals per group. Though PEPCK-bGH mice are phenotypically distinct, being large from their normal littermates, tissue genotyping was done via normal PCR and qPCR, and the presence of the bovine growth hormone gene was confirmed. The PCR products of the bGH gene, with a size of 446 bp, were amplified using bGH primers. The DNA amplicons were detected in 1.5% agarose gel following electrophoresis for about 1 hour and visualized under UV light.

### Body weight measurements

Measurement of body weight was done once a week, starting at the 12th week to the 40th week.

### Normal diet and Gly-Low diet composition

Mice were fed either a standard low-fat chow diet (21% fat (kcal), 60% carbohydrate (kcal) Envigo: TD.200743), a standard high fat chow diet (60% fat (kcal), 21% carbohydrate (kcal), Envigo: TD.200299), a standard low-fat chow diet supplemented with our Gly-Low compound cocktail (21% fat (kcal), 60% carbohydrate (kcal) Envigo: TD.200742), or a standard high fat chow diet supplemented with our Gly-Low compound cocktail (60% fat (kcal), 21% carbohydrate (kcal).

A combination of supplemental grade compounds, safe to be consumed in set dosages, were prepared and incorporated into a modified pre-irradiated standard AIN-93G mouse chow diet from Envigo. The cocktail consists of alpha lipoic acid (20.19%), nicotinamide (57.68%), thiamine hydrochloride (4.04%), piperine (1.73%), and pyridoxamine dihydrochloride (16.36%), and is supplemented in the diet to achieve a daily consumption rate in mg/kg of body weight/day. For diet, these percentages translate to 3 g/kg alpha lipoic acid, 8.57 g/kg nicotinamide, 0.6 g/kg thiamine hydrochloride, 0.26 g/kg piperine, and 2.43 g/kg pyridoxamine dihydrochloride.

### Insulin tolerance test

Insulin sensitivity test was done at two time points, 19th and 37th weeks of mice age, i.e., 9 and 27 weeks after starting the Gly-Low treatment via tail bleed in non-fasted mice. Insulin (Sigma, St. Louis, MO, USA) was injected intraperitoneally at one international unit (IU) per kg body weight. Blood glucose was measured at 0, 15, 30, 45, 60, and 120 minutes via glucometer (Agal Matrix). Data from the ITT are shown as a percentage of baseline glucose and as an area under the curve.

### Glucose tolerance test

One week after ITT, a glucose tolerance test was done at two-time points, the 20th and 38th weeks of mice age, i.e., 10 and 28 weeks after starting the Gly-Low treatment via tail bleed. All mice were fasted overnight for 16 hours before the glucose injection. Glucose was injected via intraperitoneal injection at 2 grams/kg body weight. Blood glucose was measured at 0, 15, 30, 45, 60, and 120 minutes via glucometer (Agal Matrix). Data from the GTT are shown as a percentage of baseline glucose and as an area under the curve.

### Rotarod

A rotarod test was done to assess the neuromuscular balance or motor coordination at week 39. Mice were trained to run on a rotating wheel for 5 minutes two times on day 1, and an actual run was done on day 2 to calculate the average latency for falling.

### Grip-strength

Grip strength test was done on 39–40th weeks to determine the muscular strength generated by forearm muscle in 3 trials on the same day. The body weight normalized all grip strength values.

### Tissue collection

All mice were euthanized between 9 AM and 11 AM during the 40 weeks of age or 30th week after treatment to collect the blood and tissues, including liver, muscle, pancreas, colon, heart, brain, hypothalamus, hippocampus, subcutaneous, epididymal, brown and peri-renal fat. Isoflurane was used to anesthetize the animals before being bled by heart puncture and decapacitated. During the collection, tissues were frozen in dry ice and stored at −80°C.

### RNA extraction and cDNA synthesis

Total RNA was extracted utilizing the Trizol method, and cDNA was produced via reverse transcription. Two-step real-time PCR system was used to perform SYBR green-based real-time reverse transcriptase PCR. The housekeeping gene was selected based on the type of tissue used to normalize values for the genes of interest, and the relative amounts of mRNA were calculated using the Ct technique.

### RNA sequencing

RNA was isolated using Zymo research quick RNA miniprep kit (cat # 11–328) according to the manufacturer’s recommendations. Isolated RNA was sent for library preparation and sequencing by Novogene Corporation Inc. where RNA was poly-A selected using poly-T oligo-attached magnetic beads, fragmented, reverse transcribed using random hexamer primers followed by second strand cDNA synthesis using dTTP for non-directional library preparation. Samples underwent end repair, A-tailing, adapter ligated, size selected, amplified, and purified. Illumina libraries were quantified using Qubit and qPCR and analyzed for size distribution using a bioanalyzer. Libraries were pooled and sequenced on an Illumina Novoseq 6000 to acquire paired-end 150 bp reads. Data quality was assessed, and adaptor reads, and low-quality reads were removed. Reads that passed the quality filtering process were mapped paired end to the reference genome (GRCm38) using Hisat2 v2.0.5. feature Counts v1.5.0-p3 was used to count reads that mapped to each gene. Differential expression analysis was performed using DESEq2 (1.20.0). Where indicated, bootstrapping was performed using R (R version 4.1.2) program ‘boot’ (1.3–28.1). To determine the expected mean and standard deviation, *n* = i log2 fold changes were randomly selected 1000 times, in which is the number of genes in the gene set.

### Metabolite extraction

Metabolite extraction from the mice liver tissues was performed using a modified Bligh-Dyer method. Briefly, to about 10 mg of liver tissue, 600 μl of pre-chilled methanol: chloroform (2:1) was added and tissues were homogenized. This was followed by addition of 200 μl each of water and chloroform. The samples were vortexed and centrifuged for 10 minutes at 18000 g for 10 minutes. From each sample, 350 μl of upper fraction containing polar metabolites were collected, dried using a vacuum concentrator operated at room temperature and stored in −80°C freezers till mass spectrometry runs. Samples were reconstituted in 100 μl of acetonitrile: water (50:50) and transferred to HPLC vials before acquisition on the LC-MS system.

### Mass spectrometry

The metabolomics data was acquired using a multiple reaction monitoring (MRM) method on an Agilent 6495C triple quadrupole mass spectrometer coupled to 1290 Infinity II liquid chromatography system. Optimized MRM parameters were used (as mentioned in the Table), and data acquisition was performed through Agilent Mass Hunter software in positive ion scan mode. 10 μl of metabolites suspension was loaded and resolved in a 15-minute-long gradient program on an Acquity UPLC BEH HILIC column (1.7 μm, 2.1 × 100 mm, Waters), with a flow rate of 0.3 ml/min. The column oven temperature was set at 35°C. The mobile phases comprise of 20 mM ammonium acetate and 0.1% formic acid (buffer A) and 95% acetonitrile with 0.1% formic acid (buffer B). The linear mobile phase was applied from 5% to 50% of buffer A. The gradient program was used as follows: 95% buffer B for 0–1 minute, 95–65% buffer B in the next 8 minutes, and 65% to 50% buffer B in the next 2 minutes, buffer B was changed from 50% to initial concentration of 95% in the next 0.5 minute, and the column was conditioned at the same buffer composition for 1.5 minutes. Data was acquired in four biological replicates, and the relative quantification was performed using Skyline software (v25.1) [48].

### Protein analysis

For circulating proteins, serum sample was taken during the sacrifice and centrifuged to get plasma. According to the manufacturer’s instructions, ELISA was used to quantify the serum levels of IGF-1, and insulin (Crystal Chem Inc., Elk Grove Village, IL, USA).

For protein expression, liver tissue (WT, bGH-Tg, and bGH-Tg + Gly-Low) was homogenized on ice in RIPA buffer (50 mM Tris-HCl pH 7.4, 150 mM NaCl, 1% NP-40, 0.5% sodium deoxycholate, 0.1% SDS) supplemented with protease and phosphatase inhibitor cocktails. Lysates were cleared by centrifugation (12,000 g, 10 min, 4°C) and protein concentration was quantified by BCA assay. Equal amounts of protein (as indicated in figure legends) were denatured in 4× Laemmli buffer with β-mercaptoethanol at 95°C for 5 min, separated by SDS-PAGE (8–12% gels chosen according to target molecular weight), and transferred to PVDF membranes. Membranes were blocked for 1 h at room temperature in 5% non-fat milk (TBST; 20 mM Tris-HCl pH 7.6, 150 mM NaCl, 0.1% Tween-20) and incubated overnight at 4°C with primary antibodies diluted in 5% BSA/TBST as follows: SREBF1 (Proteintech, USA, 14088-1-AP; rabbit; expected ~125 kDa; 1:1000), CLOCK (Cell Signaling Technology, USA, D45B10; rabbit; ~100 kDa; 1:1000), PPAR-α (Thermo Fisher, USA, PA1-822A; rabbit; ~68 kDa; 1:1000), and β-actin loading control (Cell Signaling Technology, 13E5; rabbit; ~45 kDa; 1:1000). After three 10-min TBST washes, membranes were incubated with HRP-conjugated goat anti-rabbit IgG secondary antibody (Cell Signaling Technology, #7074; 1:5000 in 5% milk/TBST) for 1 h at room temperature, washed, and developed using enhanced chemiluminescence. Chemiluminescent signals were captured on a digital imager with exposures within the linear range. Band intensities were quantified in ImageJ/Fiji; target protein signals were normalized to β-actin from the same membrane, and data were analyzed as described in the Statistics section. Expected single major bands were observed at the indicated molecular weights for each target; any lanes with non-specific bands or saturation were excluded a priori according to predefined QA criteria. Replicate numbers and exact protein loads per lane are reported in the corresponding figure legends.

### Quantification of Di-carbonyls

200 μL of 80:20 MeOH:ddH_2_O (−80°C) containing 50 pmol ^13^C_3_-MGO was added to 10 μL of serum and extracted at −80°C overnight. Insoluble protein was removed via centrifugation at 14,000 × g for 10 min at 4°C. Supernatants were derivatized with 10 μL of 10 mM o-phenylenediamine for 2 h with end-over-end rotation protected from light. Derivatized samples were centrifuged at 14,000 × *g* for 10 min, and the supernatant was chromatographed using a Shimadzu LC system equipped with a 150 × 2 mm, 3 μm particle diameter Luna C_18_ column (Phenomenex, Torrance, CA, USA) at a flow rate of 0.450 mL/min. Buffer A (0.1% formic acid in H_2_O) was held at 90% for 0.25 min, then a linear gradient to 98% solvent B (0.1% formic acid in acetonitrile) was applied over 4 min. The column was held at 98% B for 1.5 min, washed at 90% A for 0.5 min, and equilibrated to 99% A for 2 min. Multiple reaction monitoring (MRM) was conducted in positive ion mode using an AB SCIEX 4500 QTRAP with the following transitions: *m/z* 145.1→77.1 (MGO); *m/z* 235.0→157.0 (3-DG); m/z 131.0→77.0 (GO); m/z 161.0→77.0 (HPA); m/z 148.1→77.1 (^13^C_3_-MGO, internal standard).

### Statistical analysis

The data are shown as mean ± SEM. GraphPad Prism Software V8.0 was used for the analysis. A student’s *t*-test was used to compare the means of the two groups. The significance of the differences in the insulin tolerance test (ITT) and glucose tolerance test (GTT), indirect calorimetry was calculated by comparing the area under the curve (AUC). Two-way or three-way ANOVA with Tukey’s post hoc tests were used to compare the mean for more than two groups. *P*-values less than 0.05 were considered statistically significant. Significant differences are indicated: ^∗^*p* ≤ 0.05, ^∗∗^*p* ≤ 0.005, ^∗∗∗^*p* ≤ 0.0005, ^∗∗∗∗^*p* < 0.0001.

### Data availability

The data supporting this study’s findings are available from the corresponding author upon request.

## Supplementary Materials

Supplementary Figures

## References

[1] Morgan SA, Berryman DE, List EO, Lavery GG, Stewart PM, Kopchick JJ. Regulation of 11β-HSD1 by GH/IGF-1 in key metabolic tissues may contribute to metabolic disease in GH deficient patients. Growth Horm IGF Res. 2022; 62:101440. 10.1016/j.ghir.2021.101440 34814007

[2] Balasubramanian P, Longo VD. Growth factors, aging and age-related diseases. Growth Horm IGF Res. 2016; 28:66–8. 10.1016/j.ghir.2016.01.001 26883276 PMC5455771

[3] Lubbers ER, Magon V, Jara A, Ziegler AK, Miles B, List EO, Kopchick JJ, Berryman DE. P02-25 Adipokine levels in mice with altered Growth Hormone (GH) action over lifespan: Identifying the links between adiposity, insulin sensitivity and longevity. Growth Horm IGF Res. 2012; 22:S62. 10.1016/S1096-6374(12)60163-4

[4] Donato J Jr, Wasinski F, Furigo IC, Metzger M, Frazão R. Central Regulation of Metabolism by Growth Hormone. Cells. 2021; 10:129. 10.3390/cells10010129 33440789 PMC7827386

[5] Lopes-Pinto M, Marques PL, Lacerda-Nobre E, Miceli D, Leal RO, Marques P. Acromegaly in humans and cats: Pathophysiological, clinical and management resemblances and differences. Growth Horm IGF Res. 2024; 76:101595. 10.1016/j.ghir.2024.101595 38810595

[6] Donoho DA, Bose N, Zada G, Carmichael JD. Management of aggressive growth hormone secreting pituitary adenomas. Pituitary. 2017; 20:169–78. 10.1007/s11102-016-0781-7 27987061

[7] Osganian SA, Subudhi S, Masia R, Drescher HK, Bartsch LM, Chicote ML, Chung RT, Gee DW, Witkowski ER, Bredella MA, Lauer GM, Corey KE, Dichtel LE. Expression of IGF-1 receptor and GH receptor in hepatic tissue of patients with nonalcoholic fatty liver disease and nonalcoholic steatohepatitis. Growth Horm IGF Res. 2022; 65:101482. 10.1016/j.ghir.2022.101482 35780715 PMC9885486

[8] Olsson B, Bohlooly-Y M, Fitzgerald SM, Frick F, Ljungberg A, Ahrén B, Törnell J, Bergström G, Oscarsson J. Bovine growth hormone transgenic mice are resistant to diet-induced obesity but develop hyperphagia, dyslipidemia, and diabetes on a high-fat diet. Endocrinology. 2005; 146:920–30. 10.1210/en.2004-1232 15539551

[9] Cheriathundam E, Doi SQ, Knapp JR, Jasser MZ, Kopchick JJ, Alvares AP. Consequences of overexpression of growth hormone in transgenic mice on liver cytochrome P450 enzymes. Biochem Pharmacol. 1998; 55:1481–7. 10.1016/s0006-2952(97)00667-9 10076541

[10] Miquet JG, Freund T, Martinez CS, González L, Díaz ME, Micucci GP, Zotta E, Boparai RK, Bartke A, Turyn D, Sotelo AI. Hepatocellular alterations and dysregulation of oncogenic pathways in the liver of transgenic mice overexpressing growth hormone. Cell Cycle. 2013; 12:1042–57. 10.4161/cc.24026 23428905 PMC3646861

[11] Reynolds CM, Li M, Gray C, Vickers MH. Preweaning growth hormone treatment ameliorates adipose tissue insulin resistance and inflammation in adult male offspring following maternal undernutrition. Endocrinology. 2013; 154:2676–86. 10.1210/en.2013-1146 23715866

[12] Sadagurski M, Landeryou T, Cady G, Kopchick JJ, List EO, Berryman DE, Bartke A, Miller RA. Growth hormone modulates hypothalamic inflammation in long-lived pituitary dwarf mice. Aging Cell. 2015; 14:1045–54. 10.1111/acel.12382 26268661 PMC4693470

[13] Masternak MM, Bartke A. Growth hormone, inflammation and aging. Pathobiol Aging Age Relat Dis. 2012; 2. 10.3402/pba.v2i0.17293 22953033 PMC3417471

[14] Oeckinghaus A, Ghosh S. The NF-kappaB family of transcription factors and its regulation. Cold Spring Harb Perspect Biol. 2009; 1:a000034. 10.1101/cshperspect.a000034 20066092 PMC2773619

[15] Li X, Li C, Zhang W, Wang Y, Qian P, Huang H. Inflammation and aging: signaling pathways and intervention therapies. Signal Transduct Target Ther. 2023; 8:239. 10.1038/s41392-023-01502-8 37291105 PMC10248351

[16] Roy AK, Oh T, Rivera O, Mubiru J, Song CS, Chatterjee B. Impacts of transcriptional regulation on aging and senescence. Ageing Res Rev. 2002; 1:367–80. 10.1016/s1568-1637(02)00006-5 12067592

[17] Ogrodnik M, Miwa S, Tchkonia T, Tiniakos D, Wilson CL, Lahat A, Day CP, Burt A, Palmer A, Anstee QM, Grellscheid SN, Hoeijmakers JHJ, Barnhoorn S, et al. Cellular senescence drives age-dependent hepatic steatosis. Nat Commun. 2017; 8:15691. 10.1038/ncomms15691 28608850 PMC5474745

[18] Ogrodnik M, Carlos Acosta J, Adams PD, d'Adda di Fagagna F, Baker DJ, Bishop CL, Chandra T, Collado M, Gil J, Gorgoulis V, Gruber F, Hara E, Jansen-Dürr P, et al. Guidelines for minimal information on cellular senescence experimentation in vivo. Cell. 2024; 187:4150–75. 10.1016/j.cell.2024.05.059 39121846 PMC11790242

[19] Cordoba-Chacon J, Majumdar N, List EO, Diaz-Ruiz A, Frank SJ, Manzano A, Bartrons R, Puchowicz M, Kopchick JJ, Kineman RD. Growth Hormone Inhibits Hepatic De Novo Lipogenesis in Adult Mice. Diabetes. 2015; 64:3093–103. 10.2337/db15-0370 26015548 PMC4542445

[20] Bravo-Ruiz I, Medina MÁ, Martínez-Poveda B. From Food to Genes: Transcriptional Regulation of Metabolism by Lipids and Carbohydrates. Nutrients. 2021; 13:1513. 10.3390/nu13051513 33946267 PMC8145205

[21] Karagianni P, Talianidis I. Transcription factor networks regulating hepatic fatty acid metabolism. Biochim Biophys Acta. 2015; 1851:2–8. 10.1016/j.bbalip.2014.05.001 24814048

[22] Raghow R, Yellaturu C, Deng X, Park EA, Elam MB. SREBPs: the crossroads of physiological and pathological lipid homeostasis. Trends Endocrinol Metab. 2008; 19:65–73. 10.1016/j.tem.2007.10.009 18291668

[23] Chakravarthy MV, Pan Z, Zhu Y, Tordjman K, Schneider JG, Coleman T, Turk J, Semenkovich CF. "New" hepatic fat activates PPARalpha to maintain glucose, lipid, and cholesterol homeostasis. Cell Metab. 2005; 1:309–22. 10.1016/j.cmet.2005.04.002 16054078

[24] Hussain MM, Pan X. Clock genes, intestinal transport and plasma lipid homeostasis. Trends Endocrinol Metab. 2009; 20:177–85. 10.1016/j.tem.2009.01.001 19349191 PMC4544755

[25] Suwała S, Junik R. Metabolic-associated fatty liver disease and the role of hormones in its aetiopathogenesis. Endokrynol Pol. 2024; 75:237–52. 10.5603/ep.99689 38923899

[26] Von-Hafe M, Borges-Canha M, Vale C, Leite AR, Sérgio Neves J, Carvalho D, Leite-Moreira A. Nonalcoholic Fatty Liver Disease and Endocrine Axes-A Scoping Review. Metabolites. 2022; 12:298. 10.3390/metabo12040298 35448486 PMC9026925

[27] Baechle JJ, Chen N, Makhijani P, Winer S, Furman D, Winer DA. Chronic inflammation and the hallmarks of aging. Mol Metab. 2023; 74:101755. 10.1016/j.molmet.2023.101755 37329949 PMC10359950

[28] Weinhage T, Wirth T, Schütz P, Becker P, Lueken A, Skryabin BV, Wittkowski H, Foell D. The Receptor for Advanced Glycation Endproducts (RAGE) Contributes to Severe Inflammatory Liver Injury in Mice. Front Immunol. 2020; 11:1157. 10.3389/fimmu.2020.01157 32670276 PMC7326105

[29] Christidis G, Küppers F, Karatayli SC, Karatayli E, Weber SN, Lammert F, Krawczyk M. Skin advanced glycation end-products as indicators of the metabolic profile in diabetes mellitus: correlations with glycemic control, liver phenotypes and metabolic biomarkers. BMC Endocr Disord. 2024; 24:31. 10.1186/s12902-024-01558-9 38443880 PMC10913560

[30] Basta G, Del Turco S, Navarra T, Lee WM, and Acute Liver Failure Study Group. Circulating levels of soluble receptor for advanced glycation end products and ligands of the receptor for advanced glycation end products in patients with acute liver failure. Liver Transpl. 2015; 21:847–54. 10.1002/lt.24129 25825217 PMC4933521

[31] Chaudhuri J, Bains Y, Guha S, Kahn A, Hall D, Bose N, Gugliucci A, Kapahi P. The Role of Advanced Glycation End Products in Aging and Metabolic Diseases: Bridging Association and Causality. Cell Metab. 2018; 28:337–52. 10.1016/j.cmet.2018.08.014 30184484 PMC6355252

[32] Brownlee M. Advanced protein glycosylation in diabetes and aging. Annu Rev Med. 1995; 46:223–34. 10.1146/annurev.med.46.1.223 7598459

[33] Zunkel K, Simm A, Bartling B. Long-term intake of the reactive metabolite methylglyoxal is not toxic in mice. Food Chem Toxicol. 2020; 141:111333. 10.1016/j.fct.2020.111333 32298726

[34] Wimer L, Kaneshiro KR, Ramirez J, Valdearcos M, Shanmugam M, Gaffney D, Singh P, Beck J, Sellegounder D, Cho SJ, Newman J, Galligan J, Koliwad S, et al. Combination therapy of glycation lowering compounds reduces caloric intake, improves insulin sensitivity, and extends lifespan. bioRxiv. 2022. 10.1101/2022.08.10.503411

[35] Zeng L, Lu M, Mori K, Luo S, Lee AS, Zhu Y, Shyy JYJ. ATF6 modulates SREBP2-mediated lipogenesis. EMBO J. 2008; 27:2941. 10.1038/emboj.2008.225 PMC381012 14765107

[36] Moncan M, Mnich K, Blomme A, Almanza A, Samali A, Gorman AM. Regulation of lipid metabolism by the unfolded protein response. J Cell Mol Med. 2021; 25:1359–70. 10.1111/jcmm.16255 33398919 PMC7875919

[37] Challet E, Vuillez P. Circadian clocks and metabolism. Chronobiology and Chronomedicine. RSC. 2024; 476–504. 10.1039/BK9781839167553-00476

[38] Coomans CP, Lucassen EA, Kooijman S, Fifel K, Deboer T, Rensen PC, Michel S, Meijer JH. Plasticity of circadian clocks and consequences for metabolism. Diabetes Obes Metab. 2015 (Suppl 1); 17:65–75. 10.1111/dom.12513 26332970

[39] Sancar G, Brunner M. Circadian clocks and energy metabolism. Cell Mol Life Sci. 2014; 71:2667–80. 10.1007/s00018-014-1574-7 24515123 PMC11113245

[40] Erol A. The Functions of PPARs in Aging and Longevity. PPAR Res. 2007; 2007:39654. 10.1155/2007/39654 18317516 PMC2254525

[41] Wolf E, Kahnt E, Ehrlein J, Hermanns W, Brem G, Wanke R. Effects of long-term elevated serum levels of growth hormone on life expectancy of mice: lessons from transgenic animal models. Mech Ageing Dev. 1993; 68:71–87. 10.1016/0047-6374(93)90141-d 8350664

[42] Bartke A, Brown-Borg HM, Bode AM, Carlson J, Hunter WS, Bronson RT. Does growth hormone prevent or accelerate aging? Exp Gerontol. 1998; 33:675–87. 10.1016/s0531-5565(98)00032-1 9951615

[43] Brown-Borg HM. Growth Hormone, Not IGF-1 Is the Key Longevity Regulator in Mammals. J Gerontol A Biol Sci Med Sci. 2022; 77:1719–23. 10.1093/gerona/glac092 35436323 PMC9434454

[44] Basu R, Qian Y, Kopchick JJ. MECHANISMS In ENDOCRINOLOGY: Lessons from growth hormone receptor gene-disrupted mice: are there benefits of endocrine defects? Eur J Endocrinol. 2018; 178:R155–81. 10.1530/EJE-18-0018 29459441

[45] Bartke A. Somatotropic Axis, Pace of Life and Aging. Front Endocrinol (Lausanne). 2022; 13:916139. 10.3389/fendo.2022.916139 35909509 PMC9329927

[46] Aguiar-Oliveira MH, Bartke A. Growth Hormone Deficiency: Health and Longevity. Endocr Rev. 2019; 40:575–601. 10.1210/er.2018-00216 30576428 PMC6416709

[47] McGrane MM, Yun JS, Moorman AF, Lamers WH, Hendrick GK, Arafah BM, Park EA, Wagner TE, Hanson RW. Metabolic effects of developmental, tissue-, and cell-specific expression of a chimeric phosphoenolpyruvate carboxykinase (GTP)/bovine growth hormone gene in transgenic mice. J Biol Chem. 1990; 265:22371–9. 1702419

[48] Adams KJ, Pratt B, Bose N, Dubois LG, St John-Williams L, Perrott KM, Ky K, Kapahi P, Sharma V, MacCoss MJ, Moseley MA, Colton CA, MacLean BX, et al, and Alzheimer’s Disease Metabolomics Consortium. Skyline for Small Molecules: A Unifying Software Package for Quantitative Metabolomics. J Proteome Res. 2020; 19:1447–58. 10.1021/acs.jproteome.9b00640 31984744 PMC7127945

