# Supplementary material for: Growth hormone excess drives liver aging via increased glycation stress
Source: Aging (Albany NY). 2025 Oct 3;17(10):2534–51. doi: 10.18632/aging.206327 (PMC12606965; doi:10.18632/aging.206327)
Supplement: Supplementary Figures [file aging-17-10-206327-s001.pdf]

SUPPLEMENTARY FIGURES

A MCODE analysis of upregulaged DEGs

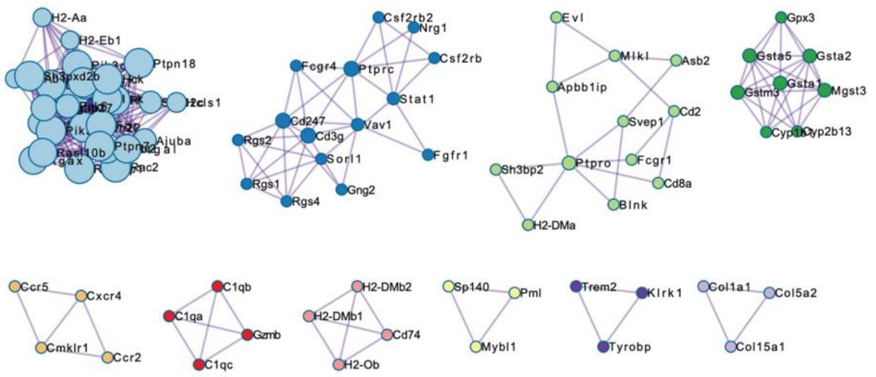

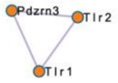

Color	MCODE	GO	Description	Log10(P)
	MCODE_1	WP85	Focal adhesion	-13.5
	MCODE_1	GO:1903039	positive regulation of leukocyte cell-cell adhesion	-9.8
	MCODE_1	GO:0022409	positive regulation of cell-cell adhesion	-9.2
	MCODE_2	R-MMU-2029482	Regulation of actin dynamics for phagocytic cup formation	-7.3
	MCODE_2	R-MMU-202427	Phosphorylation of CD3 and TCR zeta chains	-7.2
	MCODE_2	R-MMU-2029481	FCGR activation	-7.1
	MCODE_3	mmu04640	Hematopoietic cell lineage - Mus musculus (house mouse)	-6.8
	MCODE_3	GO:0002252	immune effector process	-5.1
	MCODE_3	GO:0046649	lymphocyte activation	-4.9
	MCODE_4	mmu00480	Glutathione metabolism - Mus musculus (house mouse)	-13.5
	MCODE_4	mmu00980	Metabolism of xenobiotics by cytochrome P450 - Mus musculus (house mouse)	-13.4
	MCODE_4	mmu05204	Chemical carcinogenesis - DNA adducts - Mus musculus (house mouse)	-13.0
	MCODE_5	GO:0019886	antigen processing and presentation of exogenous peptide antigen via MHC class II	-12.1
	MCODE_5	GO:0002495	antigen processing and presentation of peptide antigen via MHC class II	-11.9
	MCODE_5	GO:0002504	antigen processing and presentation of peptide or polysaccharide antigen via MHC class II	-11.8
	MCODE_6	R-MMU-173623	Classical antibody-mediated complement activation	-10.1
	MCODE_6	GO:0098883	synapse pruning	-9.8
	MCODE_6	GO:0150146	cell junction disassembly	-9.4
	MCODE_7	GO:0007204	positive regulation of cytosolic calcium ion concentration	-8.1
	MCODE_7	WP189	GPCRs class A rhodopsin like	-7.9
	MCODE_7	GO:0006935	chemotaxis	-7.2
	MCODE_9	R-MMU-8948216	Collagen chain trimerization	-8.4
	MCODE_9	R-MMU-2022090	Assembly of collagen fibrils and other multimeric structures	-8.0
	MCODE_9	R-MMU-1442490	Collagen degradation	-7.8
	MCODE_10	R-MMU-2424491	DAP12 signaling	-8.6
	MCODE_10	R-MMU-2172127	DAP12 interactions	-7.6
	MCODE_10	GO:0002705	positive regulation of leukocyte mediated immunity	-6.1

B Gene set enrichment analysis with Tabula Muris senescence database

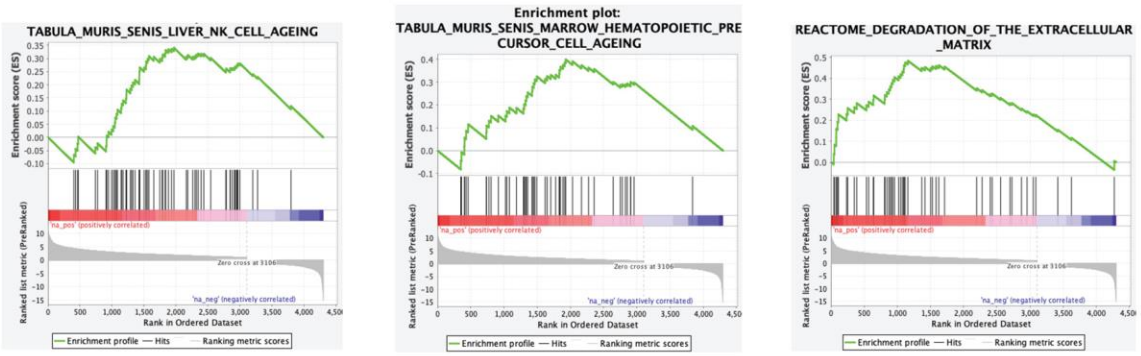

Supplementary Figure 1. Network and gene set enrichment analysis of upregulated genes in bGH-Tg liver. (**A**) MCODE analysis of upregulated DEGs identified protein–protein interaction network modules enriched in biological processes including focal adhesion, immune effector function, leukocyte cell–cell adhesion, regulation of antigen receptor signaling, and extracellular matrix organization, with each cluster color-coded and corresponding GO terms with enrichment scores (–log10 p) shown in the table below. (**B**) Gene set enrichment analysis using the Tabula Muris senescence database further highlighted significant enrichment of senescence-associated gene signatures in bGH-Tg liver, including NK cell ageing, hematopoietic precursor ageing, and extracellular matrix degradation pathways, demonstrating that GH overexpression induces transcriptional programs linked to immune activation, extracellular remodeling, and cellular senescence.

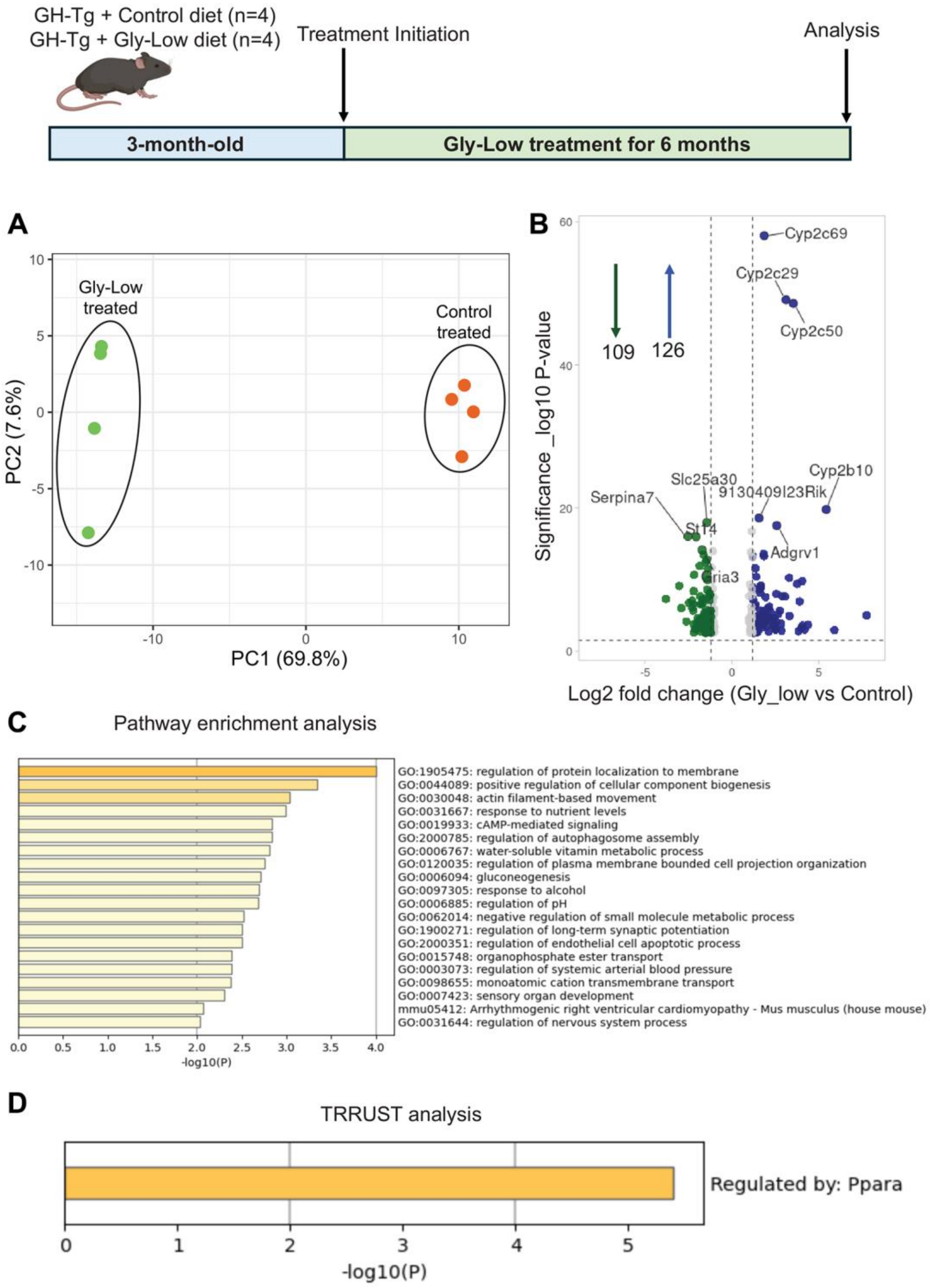

Supplementary Figure 2. Transcriptomic impact of Gly-Low treatment in the liver of bGH-Tg mice. Three-month-old WT and bGH-Tg mice were treated with either Gly-Low or control diet for 6 months, after which liver samples were collected for bulk RNA-seq analysis. Principal component analysis (**A**) shows distinct separation between Gly-Low–treated and control-treated bGH-Tg mice, indicating robust transcriptomic differences. Volcano plot analysis (**B**) identifies 235 differentially expressed genes, with 109 downregulated and 126 upregulated by Gly-Low treatment compared to control. Pathway enrichment analysis (**C**) reveals significant enrichment in processes including protein localization to the membrane, cellular component biogenesis, actin filament–based movement, cAMP-mediated signaling, and gluconeogenesis. TRRUST analysis (**D**) highlights PPARA as a key regulatory transcription factor mediating these transcriptomic changes, suggesting that Gly-Low treatment modulates metabolic and signaling pathways to mitigate GH-induced glycation stress.

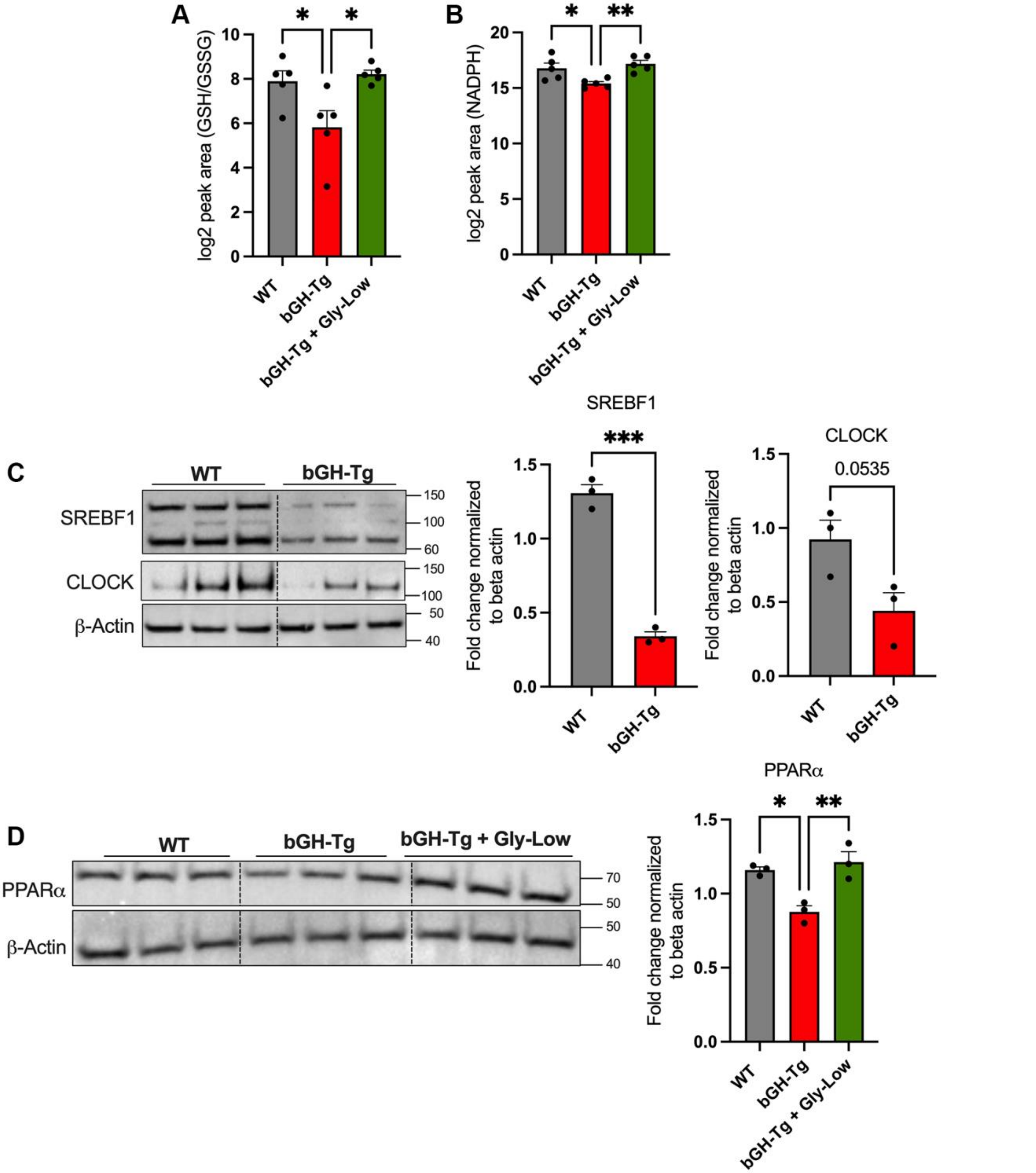

Supplementary Figure 3. Validation of oxidative stress and protein-level changes in the liver of bGH-Tg mice. (**A**, **B**) Targeted metabolomic measurements of oxidative stress markers in liver tissue. GH-Tg livers show a reduced GSH/GSSG ratio (**A**) and decreased NADPH levels (**B**), indicating increased oxidative stress. Both parameters were restored by Gly-Low treatment. (**C**) Representative Western blots and quantification of SREBF1 (~125 kDa) and CLOCK (~100 kDa) protein levels in livers from WT and bGH-Tg mice, normalized to β-actin (~45 kDa). SREBF1 was significantly reduced in GH-Tg livers, while CLOCK showed a decreasing trend. (**D**) Western blot and quantification of PPARα (~68 kDa) expression in WT, bGH-Tg, and bGH-Tg + Gly-Low livers. PPARα expression was significantly decreased in GH-Tg livers and rescued by Gly-Low treatment. Data are shown as mean ± SEM, normalized to β-actin for Western blots. Each dot represents an individual mouse. Significant differences are indicated: $^{*}p \leq 0.05$, $^{**}p \leq 0.005$, $^{***}p \leq 0.0005$.
